# Cost-utility analysis of Palbociclib + letrozole and ribociclib + letrozole versus Letrozole monotherapy in the first-line treatment of metastatic breast cancer in Iran using partitioned survival model

**DOI:** 10.1186/s13561-023-00463-6

**Published:** 2023-11-09

**Authors:** Ali Darvishi, Rajabali Daroudi, Ali Akbar Fazaeli

**Affiliations:** 1 Chronic Diseases Research Center, Endocrinology and Metabolism Population Sciences Institute, Tehran University of Medical Sciences, Tehran, Iran; 2 Department of Health Management, Policy & Economics, School of Public Health, Tehran University of Medical Sciences, Tehran, Iran; 3 Health Information Management Research Center, Tehran University of Medical Sciences, Tehran, Iran

**Keywords:** Cost-utility analysis, Palbociclib, Riboociclib, Letrozole, Metastatic breast cancer, HR+/ HER2

## Abstract

**Background:**

Palbociclib and Ribociclib are cyclin-dependent kinase 4/6 oral molecular inhibitors that have the potential to improve overall survival (OS), progression-free survival (PFS), and quality of life in patients with metastatic breast cancer (MBC). The objective of this study was to analyze the cost-utility of Palbociclib and Ribociclib in comparison with Letrozole monotherapy as the first-line treatment for hormone receptor-positive (HR+)/human epidermal growth factor receptor 2-negative (HER2–) MBC patients in Iran.

**Methods:**

A Cost-Utility Analysis (CUA) was conducted using a partitioned survival model (PSM) from the perspective of the Iranian healthcare system. The comparative strategies considered were Palbociclib + Letrozole, Ribociclib + Letrozole, and Letrozole monotherapy. The model was structured with a 1-month cycle length and a 15-year time horizon. Clinical safety, efficacy, and survival data in terms of PFS and OS for Palbociclib + Letrozole and Ribociclib + Letrozole were obtained from the latest updates of the PALOMA-1, 2, and MONALEESA-2 studies, respectively. Direct medical costs, including drug costs, visits, hospitalization, CT scans, bone x-rays, monitoring and laboratory testing, as well as medication side effects, were considered. Uncertainty evaluations were performed through deterministic sensitivity analysis and probabilistic sensitivity analysis. Excel 2016 and TreeAge 2020 were used for all stages of the evaluation.

**Results:**

The base case results indicated that, despite its lower effectiveness, Letrozole monotherapy was the most cost-effective strategy, while Palbociclib + Letrozole and Ribociclib + Letrozole were not cost-effective. The incremental cost-effectiveness ratios (ICERs) for Palbociclib + Letrozole and Ribociclib + Letrozole compared to Letrozole monotherapy were estimated at $137,302 and $120,478 per quality-adjusted life-year (QALY), respectively, which exceeded the target threshold of $4565. Deterministic sensitivity analysis demonstrated that the CUA results were not sensitive to changes in the values of uncertain variables. Probabilistic sensitivity analysis also indicated that Palbociclib + Letrozole and Ribociclib + Letrozole had no chance of being cost-effective based on changes in various parameters and simulations.

**Conclusions:**

Palbociclib and Ribociclib showed significant efficacy in combination with Letrozole, as evidenced by improvements in PFS. However, in the first-line treatment of MBC in Iran, these strategies were not cost-effective compared to Letrozole monotherapy.

## Introduction

Breast cancer is known as one of the most common cancers among women so according to World Health Organization statistics, it accounts for about 30% of cancers among women, and on average, about 2.1 million women get the disease each year [1, 2]. Studies show that this type of cancer is the second leading cause of cancer death in women in the world after lung cancer and according to 2018 estimates, 627,000 women will die from this cancer, and this number seems to be increasing [3, 4].

More than 55% of breast cancer deaths occur in low- and middle-income countries [5]. In Iran, the prevalence of this disease is reported to be 10 per 100,000 people and about 7,000 people are diagnosed annually [6].

The disease occurs in different phases in terms of prevalence and has different consequences. Most patients with metastatic breast cancer (MBC) are usually incurable with an average survival of fewer than 3 years [7]. Hormone receptor-positive (HR +) breast cancer is the most common phenotype of the disease, and patients in first-line treatment are typically treated for endocrine disorders in advanced stages consisting of aromatase inhibitors or other medicines [7, 8].

Several types of oral anticancer medicines are used to prevent breast cancer, depending on the disease stage, hormone receptors, molecular characteristics, and the general condition of the patients. These medicines are generally divided into three general categories, including chemotherapy, hormone therapy, and targeted therapy medicines [9].

Letrozole is a nonsteroidal, aromatase inhibitor, has shown efficacy in the treatment of women with early-stage or advanced, breast cancer. Letrozole is generally well tolerated and response rate, efficacy on overall survival (OS), and Progression-free survival (PFS) are significant [10].

Palbociclib is a small, reversible, and cyclin-dependent kinase oral molecular inhibitor that stops disease progression through the cell cycle [11]. PALOMA studies in phases 1 and 2, in which the drug was compared with Letrozole mono-therapy and Letrozole in combination with Palbociclib, showed a significant improvement in progression-free survival (PFS) due to Palbociclib over the average 24-months treatment period [12, 13]. However, in terms of overall survival (OS) did not show a significant effect in the phase 2 study [13]. On the other hand, improvement in PFS status was associated with increased toxicity. Also, approximately 66% of patients treated with Palbociclib experienced grade 3 and 4 neutropenia, and half of the required dose reduction [13]. On the other hand, according to some studies, the use of this medicine in patients increased their quality of life [11–14].

While there is evidence that Palbociclib may improve PFS and quality of life in MBC patients, it is very expensive and significantly increases toxicity [15, 16]. In contrast, alternative drugs such as Letrozole, although lower in terms of PFS and quality of life than Palbociclib, seems to cost less [15, 16]. In addition, according to some other studies, during the clinical development of cyclin-dependent kinase drugs, evidence of appropriate efficacy have been observed for the new drug as Ribociclib, one of the innovative and new medicines for the treatment of HR + / HER2- group of MBC [17]. Ribociclib is also in the target group of medicines similar to Pablociclib and has recently been approved. Also, other medicines such as Exemestane, Anastrozole, Fulvestrant, Abemaciclib, and even Everolimus are sometimes considered as alternative interventions for Palbociclib in various treatment lines [18]. The results of different economic evaluations in different countries, showed adding Palbociclib and Ribociclib are unlikely to be cost-effective compared to mono-therapy strategies in the first-line treatment MBC [15, 19, 20]. Also, results of comparing the cyclin-dependent kinase drugs with each other showed that Ribociclib was more cost-effective than Palbociclib [21, 22].

In general, breast cancer imposes a significant cost on the health systems. For example, the United States annually spends only more than $16 million on breast cancer treatment [23]. Although oral anti-cancer medicines for breast cancer have clinical benefits and are appropriate prescriptions, the high cost of these medicines, especially targeted therapies that have recently been approved, is a significant challenge [24, 25]. In the absence of systemic financial protection, the high financial burden may reduce the adherence of cancer patients to the treatment and thus lead to poor clinical outcomes [26].

At present, Palbociclib is not on the official Iranian drug list. Although some domestic companies have produced this drug in a limited way in recent years, in general, its main use is currently in the form of single-prescription and urgent imports. The same is true of the Ribociclib. Given the differences in clinical outcomes associated with the use of different medicines, including their safety and effectiveness, and because the cost-effectiveness of providing services is increasingly sought after in health care systems around the world, a comparative evaluation of medicines is very important in the treatment of patients with MBC.

Therefore, according to the explanations provided for the differences in the interventions’ outcomes, and also to provide appropriate evidence for deciding on the use and financing of the most appropriate clinical and economic interventions, the present study aimed to evaluate Palbociclib + Letrozole and Ribociclib + Letrozole in comparison with Letrozole mono-therapy in the first-line treatment of HR+/HER2- MBC in Iran.

## Materials and methods

### Study design

A full economic evaluation was performed using a partitioned survival model (PSM). The model was selected according to the type of interventions and target patients and the intermediate outcomes and considering MBC nature.

In this model, the lifetime cost and outcomes of treatment with alternative interventions were compared in the first line of treatment of HR+/ HER2- MBC patients.

Comparators were selected based on the latest update of guidelines and classified evidence of breast cancer treatment regimens of the National Comprehensive Cancer Network [18]. Because cyclin-dependent kinase drugs are used in combination with other drugs including aromatase inhibitors such as Letrozole in the first line of treatment, the main arm of evaluation is also considered as a combination. In general, the main intervention and its comparators are as follows:

- **ST 1:** Palbociclib + Letrozole
- **ST 2:** Ribociclib + Letrozole
- **ST 3:** Letrozole

### Modeling

To design an economic evaluation model, the models developed in previous studies were reviewed and based on the best evidence and Iran’s treatment protocols, economic modeling was performed. As mentioned, PSM was used in the present economic evaluation. The PSM is an approach to predicting state membership in cost-effectiveness models that is distinct from commonly used methods such as state transition models. State membership in PSMs can be is determined using a model structure of treatments for advanced or metastatic cancer. PSM includes 3 states included progression-free survival (PFS), progressed disease (PD). In state transition models transition between health states is based on transition probabilities and rates but in PSM movement from health states is a link to survival curves [27]. PSM initially has been used in the National Institute for Health and Care Excellence (NICE) and now is the most important approach in appraisals of MBC interventions [28].

**Fig. 1:**
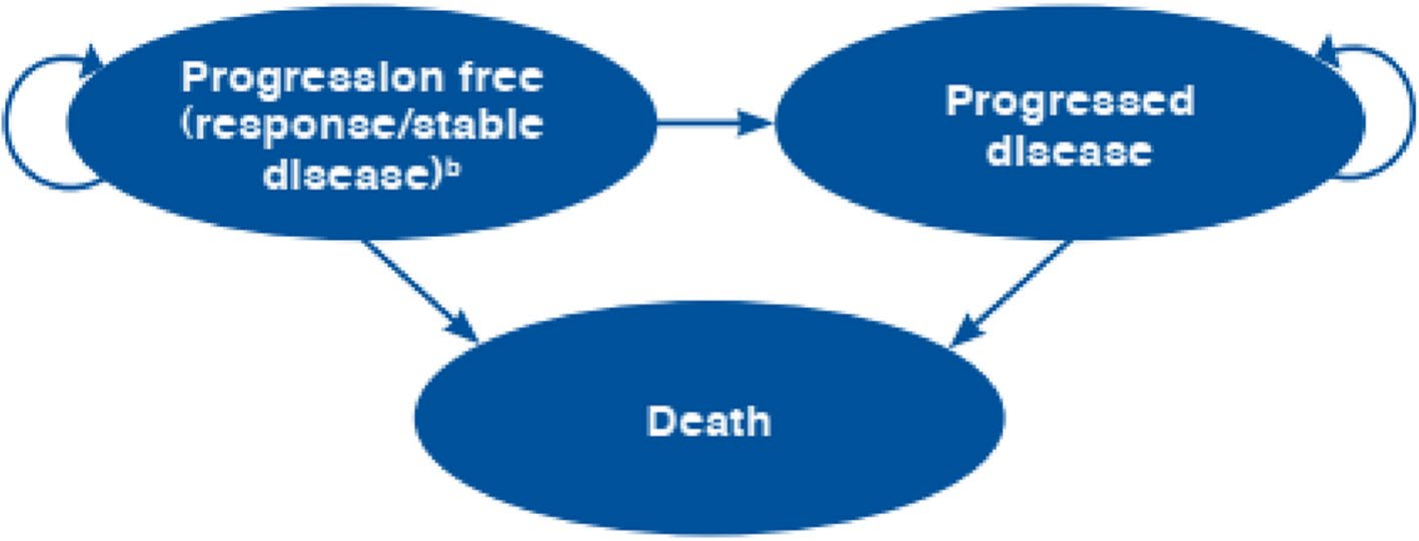
PSM structure of economic evaluation of Alternative Regimens in the treatment of HR + / HER2- MBC

The main structure of the designed model can be seen in Fig. 1. The model is structured in such a way that each strategy is based on a PSM model. In each model, 3 health states were considered included Progression-Free survival (PFS), Progressed Disease (PD) states, and Death. The modeling process in cycles is such that patients in cycle zero in the comparable groups are in a PFS health state. Individuals in each cycle are either in the state of PFS, or PD, or die, and evidence of transition probabilities was extracted according to the survival functions in each health state.

### Clinical parameters

Clinical safety and efficacy of interventions and survival functions in terms of PFS and OS for Palbociclib + Letrozole, and Ribociclib + Letrozole compared to Letrozole mono-therapy was obtained from the latest data cut-off of PALOMA-1, 2, and MONALEESA-2 study, respectively [16, 29, 30]. Regarding the efficacy of drugs and survival curves, in terms of the hazard ratio of OS, Palbociclib + Letrozole compared with Letrozole monotherapy improves the survival by about 10% [12]. Efficacy of regimens based on the PFS also showed that the Palbociclib + Letrozole compared to Letrozole mono-therapy improved PFS in patients by about 44% [12]. The median PFS was 27.6 and 14.5 months, respectively [12].

Comparing cyclin-dependent kinase drugs with each other, HR of PFS of Ribociclib + Letrozole compared with Palbociclib + Letrozole, the available evidence showed a better efficacy of Ribociclib. However, none of the values mentioned in comparing the efficacy of drugs were statistically significant [31].

**Fig. 2:**
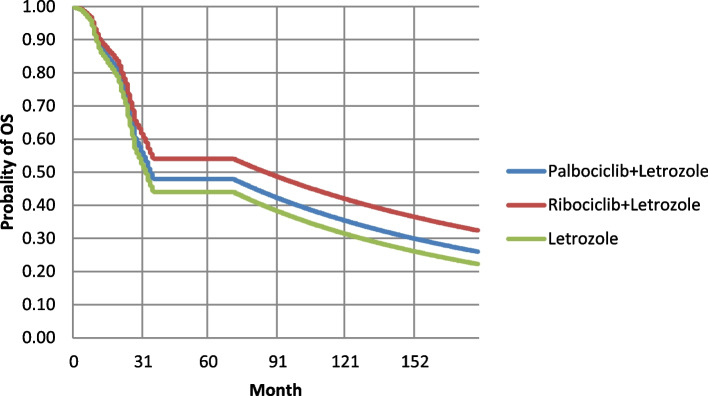
OS curves of compared strategies according to the economic evaluation time horizon in the first-line treatment of HR + / HER2- MBC

**Fig. 3:**
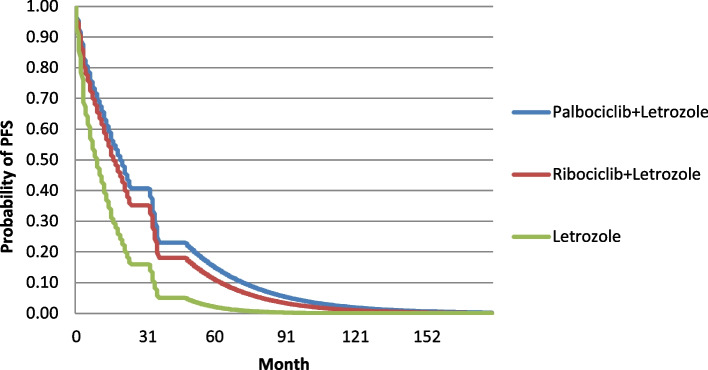
PFS curves of compared strategies according to the economic evaluation time horizon in the first-line treatment of HR + / HER2- MBC

Given that no head-to-head clinical studies were performed between Ribociclib and Palbociclib, we used the Matching-adjusted indirect comparison (MAIC) to adjust and matched individual data based mentioned studies [32]. MAIC is a technique that allows two studies to be compared when individual data are available from one study, but not from another study [32]. Considering that survival curves are used for clinical effectiveness in PSM models, in this study, according to each strategy, survival curves were extracted for as long as their data were available (latest update (cut-off) of reference clinical trial studies). in the PALOMA-1, 2, and MONALEESA-2 studies, the follow-up period was shorter than the time horizon considered in the PSM. So, the respective PFS and OS survival functions had to be extrapolated based on parametric adjustment, using Log-Normal and Weibull distributions, and adaptation to the previous trends was conducted. The values ​​of model parameters and variables and their sources can be seen in Table 1. The extrapolated PFS and OS survival curves are presented in Figs. 2 and 3.

### Model assumptions and parameter value extraction

The cost of each strategy was considered according to international evidence. Given that most clinical trial studies and international guidelines have recommended that patients receive medication until the disease progresses, the median PFS in each strategy was considered as the duration of drug cost calculation in the models.

The cycle length in PSM was considered 1 month based on the nature of the disease and interventions and the minimum time for patients to transition from one state to another, as well as evidence from past studies. This study was conducted from the perspective of the Iranian healthcare system (payer). Given that the present study is a full economic evaluation study, the outcome in this evaluation is considered quality-adjusted years life-years index (QALYs), and the cost-utility status of each strategy is ultimately estimated based on cost per QALY. Evidence of quality of life and utility in any health state has been extracted from international studies (Table 1).

In terms of costs, direct medical costs were considered according to the study perspective. These costs include the cost of drugs, the cost of periodic GP and oncologist visits, the cost of hospitalization, the cost of CT scan, bone x-rays, monitoring and testing, and the medication’s side effects. Cost items were included in the model according to health states and strategies. Details of patients’ management and monitoring costs and resource used for each of the health states are placed in Table 4 in the Appendix.

Extraction price of drugs in existing cases was done through the official website of Iran’s Food and Drug Administration. Because Palbociclib and Ribociclib drugs were not available in the list of Iranian drugs, prices were extracted based on inquiries from companies importing urgent drugs.

Other costs were extracted based on public tariffs of Iran’s Ministry of Health in 2020-21. Because the mentioned cost items are different in different MBC patients, costing was done based on clinical guidelines and also in consultation with clinical consultants.

Regarding the treatment cost of drugs complications, first, the probability of occurrence of each complication was extracted from international studies [33] and then the costing was conducted according to the common treatment strategies for each complication. Details of the cost of treatment of drugs complications and resource used for each of the strategies are placed in Table 5 in the Appendix.

### Data Analysis

The incremental cost-effectiveness ratio (ICER) was used to analyze and determine the most cost-effective strategy according to the cost and outcome of each strategy.

$$ICER=C_1-C_2/E_1-E_2$$

C represents the cost of strategies 1, 2, and E represents the amount of effectiveness of the strategies. ICER was then compared with the value of the cost-effectiveness threshold and the most cost-effective strategy was determined. According to the WHO recommendation for developing countries to select one to three times the GDP per capita as cost-effectiveness threshold, in the present study, considering that the interventions include cancer drugs and is from the end of Life Treatments, the threshold of two times Iran’s GDP per capita ($4565) in 2020 was considered as the cost-effectiveness threshold.

### Sensitivity analysis

Deterministic and Probabilistic Sensitivity Analyses was performed due to the uncertainty regarding some parameters used in the model. One-way sensitivity analysis and Tornado Diagram were used to perform deterministic sensitivity analysis. Cost of drugs, time horizon, PF, and PD health states utility values were the uncertain variables considered for deterministic sensitivity analysis. A scenario analysis was conducted considering the prices of different available brands of drugs. Also, threshold analysis was conducted to show at which drug price the other treatments would be cost-effective. Considering the probabilistic distribution of some uncertain variables, probabilistic sensitivity analysis and Monte Carlo simulation were conducted and the cost-effectiveness acceptability curve and cost-effectiveness strategy selection were estimated. The distributions of the uncertain parameters used in the probabilistic sensitivity analysis are given in Table 1. In cases where no evidence was found regarding the scattering of the uncertain variables, 20% of the mean parameter was considered as the standard deviation, and the appropriate distribution was selected according to the type of variables.

**Table 1:** Model Inputs and sources

Statistic variable	Base case	SD/(CI)	Distribution	Source
**Annual discount rate**	0.05	(0.03–0.12)	Beta	
**Time Horizon(years)**	15	(5–25)		
***Efficacy Parameters(PFS)***	***HR***			
**Palbociclib + Letrozole vs. Letrozole**	0.49	(0.32–0.75)	LogNormal	(12)
**Ribociclib + Letrozole vs. Letrozole**	0.57	(0.46–0.7)	LogNormal	(29)
***Efficacy Parameters(OS)***	***HR***			
**Palbociclib + Letrozole vs. Letrozole**	0.897	(0.623–1.294)	LogNormal	(12)
**Ribociclib + Letrozole vs. Letrozole**	0.75	(0.52–1.08)	LogNormal	(29)
***Adverse Events Costs(T)***				
**Palbociclib + Letrozole**	21.225	± 4.245	Gamma	(33),
**Letrozole**	0.775	± 0.154	Gamma	(33), Survey and Calibration
**Ribociclib + Letrozole**	23.194	± 4.638	Gamma	(33), Survey and Calibration
***Management and Monitoring Costs(T)***				
**GP Visits**	2.344			Survey and Calibration
**Oncologist Visit**	2.841			Survey and Calibration
** Computed Tomography**	9.136			Survey and Calibration
** Bone scintigraphy**	22.540			Survey and Calibration
** hospitalizations**	18.375			Survey and Calibration
** Laboratory Tests**	3.064			Survey and Calibration
***Monthly Medications Costs(T)***				
** Palbociclib (Foreign Brand 1)**	3189.911	± 637.982	Gamma	Survey
** Palbociclib (Foreign Brand 2)**	2188.427	± 437.685	Gamma	Survey
** Palbociclib (Iranian Brand)**	342.73	± 68.545	Gamma	FDA
** Letrozole (Iranian Brand)**	2.687	± 0.537	Gamma	FDA
** Letrozole (Foreign Brand)**	8.011	± 1.602	Gamma	FDA
** Ribociclib**	3938.131	± 791.543	Gamma	Survey
***Utilities***				
** PF**	0.83205	± 0.00655	Beta	(21)
** PD**	0.505	± 0.0505	Beta	(21)

### Data analysis measures

To analyze clinical efficacy data, survival curves, preparation of initial economic evaluation data including cost data and cost calculations in each health state and also other evidence, preliminary calculations, and graphs, Excel 2016 software was used. Also, modeling, analysis of base-case results in terms of cost-utility analysis as well as all stages of sensitivity analysis were performed using TreeAge 2020 software.

## Results

In this section, the findings of the economic evaluation of the study are presented in the form of two sections: Base Case and Sensitivity Analysis.

Diagrams of overall survival (OS) and progression-free survival (PFS) of the compared strategies according to the desired time horizon in economic evaluation can be seen in Figs. 2 and 3. As mentioned in the method these curves were extracted using evidence of the efficacy of strategies from related clinical trials and also was adjusted based on economic evaluation time-horizon in PSM models.

### Base case analysis

Table 2 shows the base case results of the cost-utility analysis of the comparison of the mentioned strategies in the first-line treatment of MBC. In this table, the strategies are arranged in order of 15 years average cost from low to high values, ​​and incremental cost values, incremental effectiveness as well as ICERs were calculated based on the reference strategy (lowest cost strategy). On the other hand, the net monetary benefits (NMB) of each strategy were calculated based on the amount of willingness to pay ($4565).

**Table 2:** Base case CUA of compared strategies in the first-line treatment of HR + / HER2- MBC

	Comparison Ribociclib and Palbociclib with Letrozole			Comparison between Ribociclib and Palbociclib	
Strategy	Letrozole	Palbociclib + Letrozole	Riboociclib + Letrozole	Riboociclib + Letrozole	Palbociclib + Letrozole
**Cost ($)**	7,531.34	82,041.04	96,247.45	96,247.45	82,041.04
**QALYs**	2.861	3.403	3.597	3.597	3.403
**Incremental Cost($)**		74,509.7	88,716.11	14,206.41	
**Incremental QALYs**		0.542	0.736	0.194	
**ICER($/QALY)**	(undefined)	137,302	120,478	73,342	
**NPV($)**	5,528.21	-66,504.15	-79,826.29	-79,826.29	-66,504.15

Accordingly, the results showed that despite the lower effectiveness, the Letrozole mono-therapy was the most cost-effective strategy. Therefore, the strategy of Palbociclib and Ribociclib in combination with Letrozole were not cost-effective in the first line treatment of MBC.

The ICER value of the Palbociclib + Letrozole was estimated at $137,302 per QALY compared to Letrozole alone, indicating a large interval from the target threshold amount. The ICER value of the Ribociclib + Letrozole was $120,478 per QALY compared to Letrozole mono-therapy.

As can be seen from Table 2, the NMB were 5528, -66,504, and $-79,826 for Letrozole alone, Palbociclib + Letrozole and Riboociclib + Letrozole, respectively.

Leaving aside the Letrozole mono-therapy and comparing the strategies of cyclin-dependent kinase drugs with each other, the results showed that the Palbociclib + Letrozole is in a better status in terms of cost-effectiveness than the Ribociclib + Letrozole. Also, The ICER value of the Ribociclib + Letrozole was $73,342 per QALY compared to Palbociclib + Letrozole.

### Sensitivity analysis

#### Deterministic sensitivity analysis

**Fig. 4:**
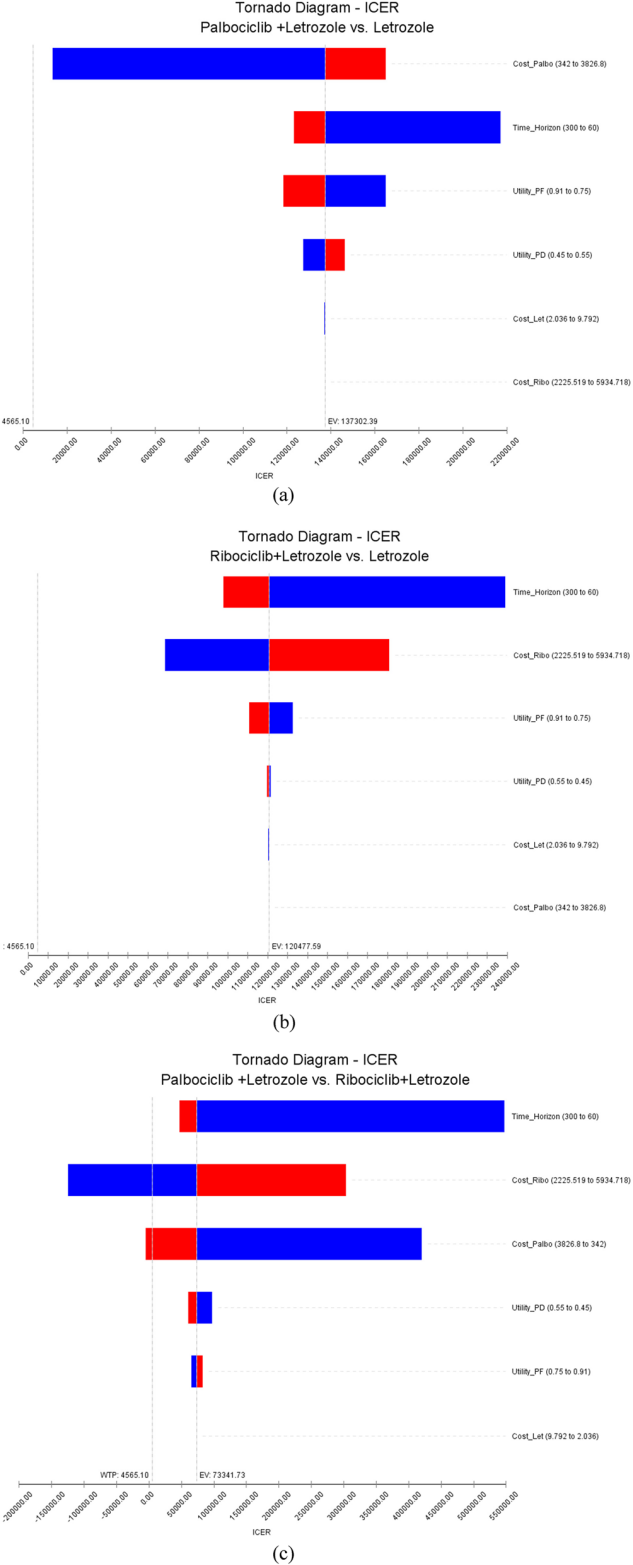
One-way sensitivity analysis of CUA of compared strategies in the first-line treatment of HR + / HER2- MBC using Tornado Diagram

In this section, according to the uncertain parameters and the confidence interval of their values, deterministic sensitivity analysis was performed. In Palbociclib + Letrozole vs. Letrozole, changes in the values of the cost of Palbociclib and time horizon had the greatest impact on the study results (Fig. 4a). In Ribociclib + Letrozole vs. Letrozole, changes in the time horizon and the cost of Ribociclib had the greatest impact on the study results (Fig. 4b), and in Palbociclib + Letrozole vs. Ribociclib + Letrozole, changes in the time horizon, cost of Ribociclib and the cost of Palbociclib had the greatest impact on the study results (Fig. 4c). But in all the changes mentioned variables did not have a threshold for cost-effectiveness, in other words, changing the values of these variables did not change the cost-effectiveness results in any way.

It should be noted that to more accurately investigate the uncertainty of the parameters in terms of impact on the results, the confidence interval of the variables was considered wide in a sensitivity analysis.

Because Palbociclib and Letrozole were available in different brands in the Iranian pharmaceutical market, which also had significant price differences, in one scenario, considering the prices of different brands of drugs, the sensitivity analysis was performed (scenario analysis) (Table 3). In this analysis, it is assumed that the effectiveness of different brands is the same and only the difference in drug cost of the different brands was taken into account. As the results showed, the Iranian Brand of Letrozole mono-therapy was cost-effective, and the results of the base case analysis in this scenario did not change, while the cost of the Iranian brand of Palbociclib is less than one-fifth of the European brand of the drug.

**Table 3:** Cost-utility analysis of compared strategies based on different Brands in the first-line treatment of HR + / HER2- MBC

Strategy	Cost ($)	Incr Cost^b^($)	Eff	Incr QALYs^b^	ICER^b^ ($/QALY)
**Letrozole (Iranian Brands)**^**a**^	7,453.69		2.861		
**Letrozole (Foreign Brands)**	7,531.34	77.65	2.861	0.000	(undefined)
**Palbociclib (Iranian Brand) + Letrozole**	14,839.08	7,385.39	3.403	0.543	13,609
**Palbociclib (Foreign Brand 2) + Letrozole**	58,403.04	50,949.35	3.403	0.543	93,887
**Palbociclib (Foreign Brand 1) + Letrozole**	82,041.04	74,587.35	3.403	0.542	137,445
**Ribociclib + Letrozole**	96,247.45	88,793.76	3.597	0.736	120,583

^a^baseline ^b^All referencing baseline

Threshold analysis was conducted to show at which drug price the other strategies would be cost-effective. The results of the threshold analysis showed that the Palbociclib (Iranian Brand) + Letrozole would be a cost-effective strategy for the $ 134.788 price of Palbociclib. Similarly Ribociclib + Letrozole would be a cost-effective strategy for the $ 110.449 price of Ribociclib.

In general, deterministic sensitivity analysis showed that the results of the CUA were not sensitive to changes in the values of uncertain variables.

#### Probabilistic sensitivity analysis

Considering the distribution function of the values of uncertain variables, probabilistic sensitivity analysis was performed using Monte Carlo Simulation by considering 1000 times of simulation repetition and sampling.

**Fig. 5:**
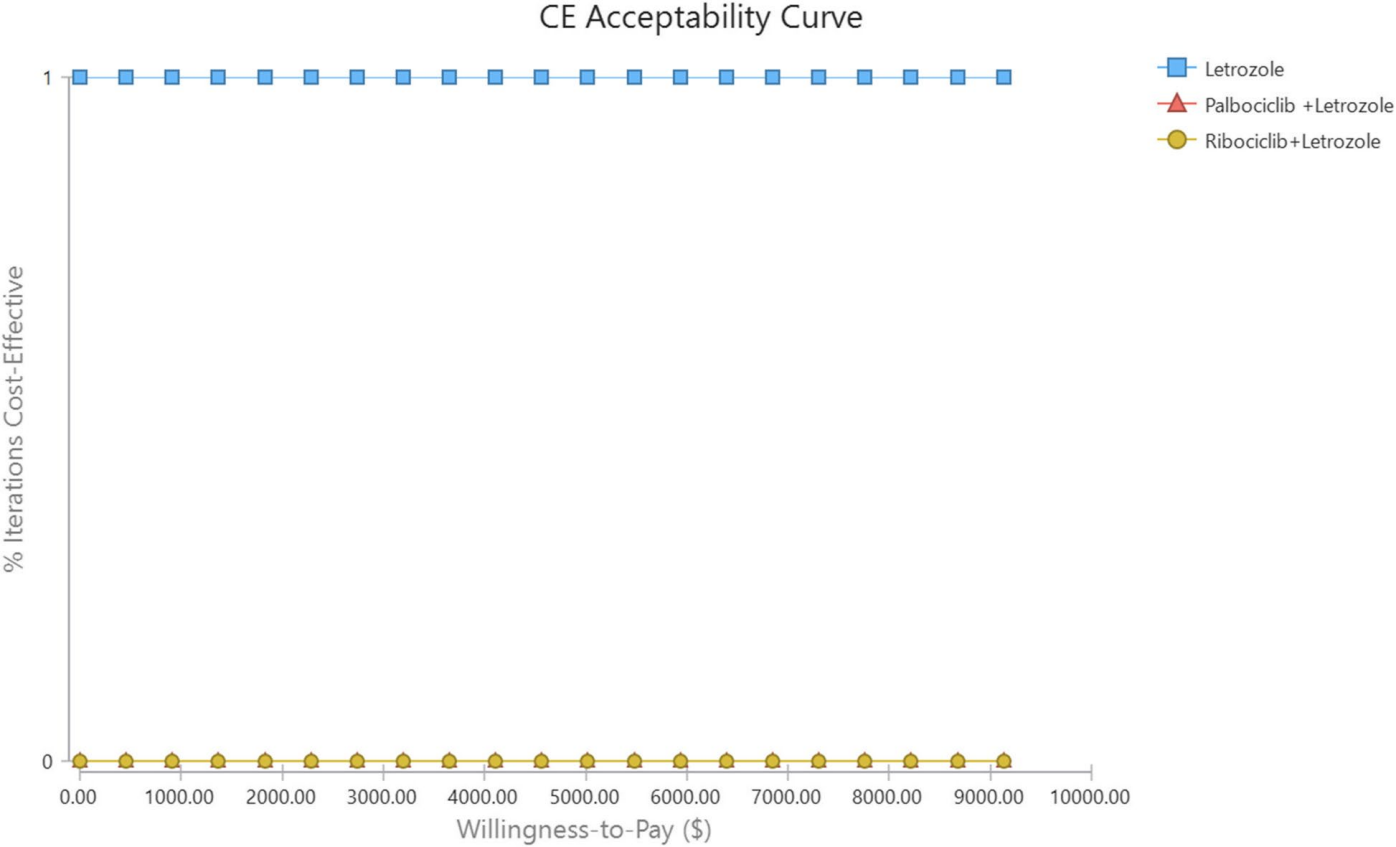
Mont-Carlo Simulation and Cost Effectiveness Acceptability Curve of CUA of compared strategies in the first-line treatment of HR + / HER2- MBC

Based on this, the Cost-Effectiveness Acceptability curve was extracted (Fig. 5). As can be seen, by increasing the value of the cost-effectiveness threshold (WTP), the probability of cost-effectiveness of strategies does not change, indicating that Letrozole alone is cost-effective in all values of WTP.

**Fig. 6:**
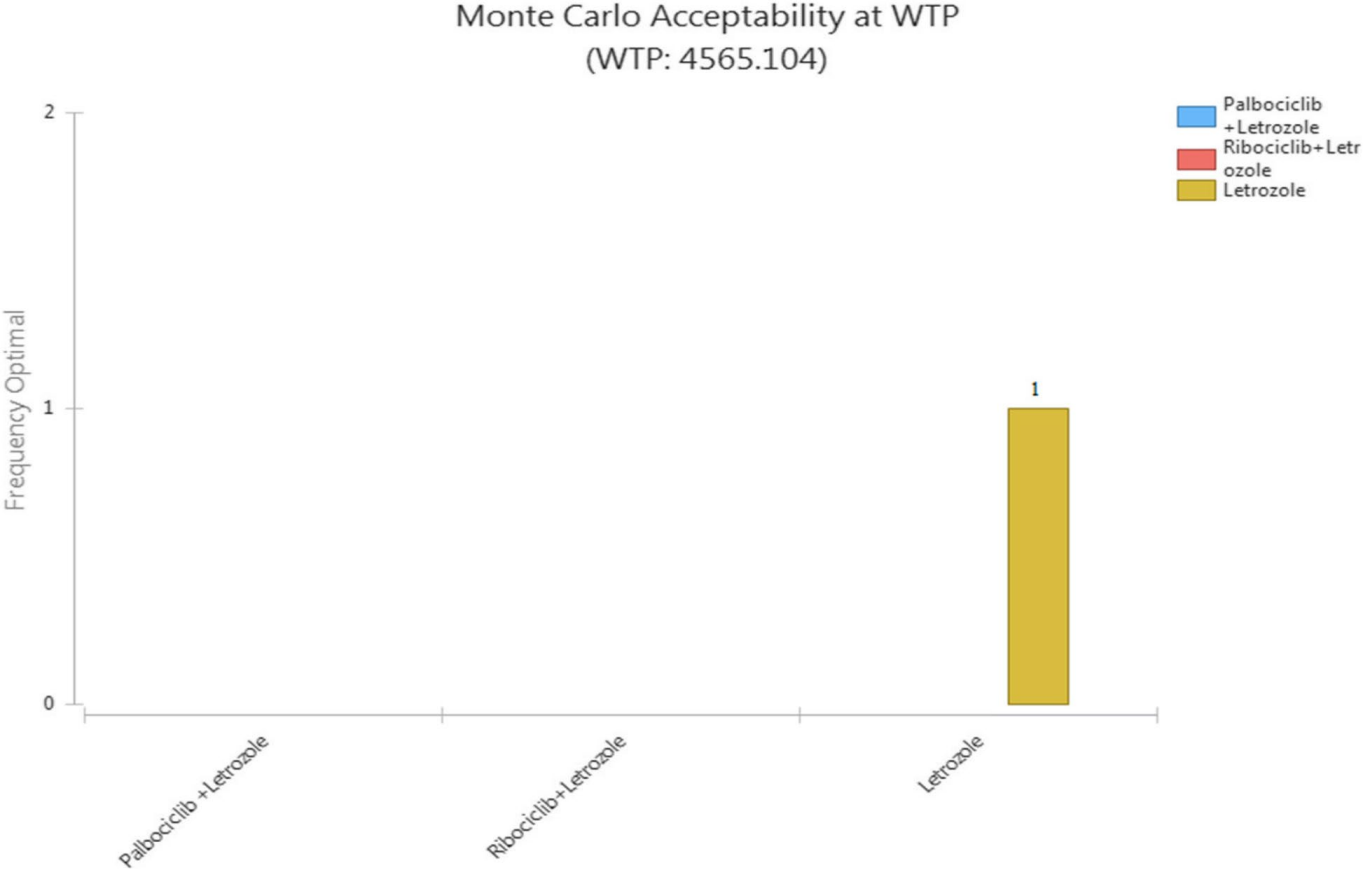
Mont-Carlo Simulation and Strategy selection diagram of CUA of compared strategies in the first-line treatment of HR + / HER2- MBC

Figure 6 (strategy selection diagram at WTP) shows the probability of optimization or, in other words, the probability of cost-effectiveness of each strategy by considering the cost-effectiveness threshold (WTP) and repetition of Monte Carlo sampling concerning the distribution of values of uncertain variables. Accordingly, as can be seen, Palbociclib + Letrozole and Riboociclib + Letrozole did not have a chance to be cost-effective based on changes in various parameters and simulations.

**Fig. 7:**
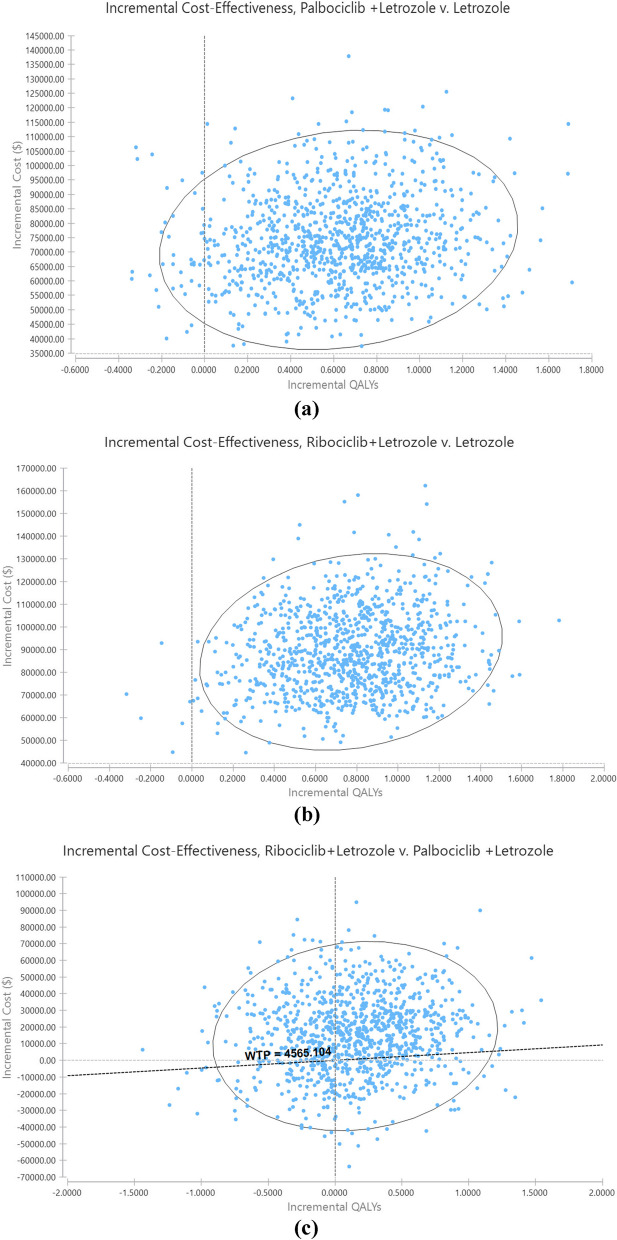
Cost-effectiveness planes of CUA of alternative regimens in the first-line treatment of HR + / HER2- MBC

Figure 7 also shows the incremental cost effectiveness scatter plots of the Palbociclib+Letrozole vs Letrozole, Riboociclib+Letrozole vs Letrozole and, Riboociclib+Letrozole vs Palbociclib+Letrozole in Monte-Carlo simulation. As can be seen in Figure 7 (a,b), in 1000 repetitions of sampling and simulations, the probability of cost-effectiveness of the Riboociclib+Letrozole and Palbociclib+Letrozole compared to Letrozole mono-therapy strategy is equal to zero. In Riboociclib+Letrozole vs Palbociclib+Letrozole, Palbociclib+Letrozole strategy is more likely to be located in cost-effectiveness quadrants and below the cost-effectiveness threshold line.

## Discussion

The present study was an economic evaluation to compare Palbiciclib versus alternative strategies in the first line of treatment of HR + / HER2- MBC in Iran. In this study, according to survival curves, the efficacy of interventions and based on PSM models, lifetime CUA was performed.

Findings of base case cost-utility analysis showed that despite lower efficacy, the Letrozole mono-therapy is the most cost-effective in the first-line treatment of MBC. Therefore, Palbociclib and Ribociclib regimens in combination with Letrozole were not cost-effective, and the ICER values were far from the intended cost-effectiveness threshold ($4565 per QALY). The results of different studies in different countries, similar to the present study, showed adding Palbociclib is highly unlikely to be cost-effective compared to mono-therapy strategies including Letrozole [15, 19, 20].

Results of comparing the strategies of cyclin-dependent kinase drugs with each other showed that Palbociclib + Letrozole was more suitable in terms of cost-effectiveness than Ribociclib + Letrozole. Compared to the present study, the results of the study by Galve-Calvo et al. (2018) in Spain showed that Ribociclib + Letrozole with more QALYs and more cost was a cost-effective strategy compared to Palbociclib + Letrozole based on the cost-effectiveness threshold of Spain [21]. Also, the findings of the Mistry et al. (2018) study in the United States showed that Ribociclib + Letrozole compared to Palbociclib + Letrozole is a dominant strategy for first-line treatment of postmenopausal women with HR + / HER2-metastatic breast cancer [22].

Regarding the sensitivity analysis of the model in evaluating the results, it showed that changes in the values ​​of uncertain variables did not have a considerable effect on the evaluation, and changing the values of uncertain variables did not change the cost-effectiveness results in any way. According to probabilistic sensitivity analysis and Monte Carlo Simulations, Palbociclib + Letrozole and Ribociclib + Letrozole had no chance of being cost-effective due to the statistical distributions. The overall results of the economic evaluation sensitivity analysis showed that the base case results of the model are highly Robust. The findings of a probabilistic sensitivity analysis in a study by Mamiya et al. (2017) in the United States also showed that from a societal perspective adding Palbociclib to the medication regimen of patients with MBC in 1st line treatment had a 0% chance of cost-effectiveness [19].

The primary limitation of this study was the inability to obtain precise pricing information for imported drugs due to fluctuating exchange rates and substantial price variations among different imported brands as well as the Iranian brand of drugs. Consequently, the pricing of drugs remained highly uncertain, and in the analysis, we attempted to address this issue by including all brands as separate strategies in the sensitivity analysis. It is important to note that, due to the lack of specific evidence, the effectiveness of all drug brands in this study was assumed to be the same. However, it is worth mentioning that the efficacy of drugs may differ across various brands and products. This analysis employed well-established clinical trial data obtained from the PALOMA-1, 2, and MONALEESA-2 studies. Another limitation of the present study was the absence of head-to-head clinical studies comparing Ribociclib and Palbociclib. To address this limitation, as previously mentioned, we utilized the MAIC method to adjust and match individual data based on the aforementioned studies.

Since we did not have internal evidence on the utility values in Iran, we tried to use the best available evidence in this regard. It is worth mentioning that, in economic evaluations, the difference between the utility values in health states is more important than the amount of utility in each health state. Therefore, given that this difference in utility values is almost the same in different contexts, the use of evidence in this regard can be justified in terms of transferability.

Our analysis provides valuable insights into Palbociclib and Ribociclib using the existing trial data, and we aim to conduct further real-world clinical studies in the future to enhance the accuracy of our economic evaluation. Additionally, exploring various other studies in this field across different treatment lines can yield diverse outcomes and contribute to a broader understanding.

## Conclusions

Palbociclib and Ribociclib showed significant efficacy in the addition to Letrozole based on the PFS. Base case and sensitivity analysis of this study showed that the Palbociclib + Letrozole and Ribociclib + Letrozole compared to Letrozole mono-therapy were not a cost-effective strategy in the first-line treatment of MBC.

There are several policy implications that can be mentioned. The high cost of Palbociclib, Ribociclib, and Letrozole may pose challenges for healthcare systems and payers. Policy interventions may be necessary to negotiate drug prices or develop reimbursement strategies that consider cost-effectiveness data, ensuring the optimal allocation of limited healthcare resources. The findings suggest that Letrozole monotherapy remains a viable and cost-effective option for the first-line treatment of MBC. It is important to ensure that healthcare professionals are aware of the cost-effectiveness data and can make informed decisions about the most appropriate treatment options. The study highlights the need for further research and development efforts to identify more cost-effective treatment options for MBC, while also providing incentives for the development of innovative therapies that demonstrate improved efficacy and cost-effectiveness. Policymakers may need to consider measures to enhance patient access to effective treatments, such as implementing assistance programs, addressing insurance coverage gaps, or exploring generic alternatives.

## Data Availability

The data that support the findings of this study are available from the corresponding author, [AF], upon reasonable request.

## Appendix

**Table 4:** Patients management and treatment costs and resource used for each of the health states

Costs attributed to health state	Progressed Disease (PD)	Progression-Free (PF)	Monthly Costs ($)	
			Progressed Disease (PD)	Progression-Free (PF)
**GP Visit**	1 visit/2 month	1 visit/2 month	1.172	1.172
**Oncologist Visit**	1 visit/ month	1 visit/ month	2.841	2.841
**Computed Tomography**	1 visit/ 3.5month	1 visit/ 3 month	2.610	3.045
**Bone scintigraphy**	1 visit/6.5 month	1 visit/6 month	3.467	3.756
**Hospitalizations**	8 days		147.002	

**Table 5:** Treatment of adverse event costs and probability of occurrence for each of strategies

Adverse Events	Monthly Total AE Costs ($)	Probability of Occurrence (%) (33)		
		Palbociclib+Letrozole	Letrozole	Ribociclib+Letrozole
**Neutropenia**	31.379	52.05	0.95	52.4
**leukopenia**	31.379	21.55	0	20.1
**Nausea**	6.743	1.1	1.4	2.4
**vomiting**	6.7433	0.25	1.2	3.6
**Diarrhea**	1.658	2.7	0.7	2.4
**Anemia**	23.661	5.5	1.2	1.2
**Pulmonary Embolism**	164.688	3.175	0.3	0.45

## Abbreviations

CUA: Cost-Utility Analysis
OS: Overall survival
ICER: Incremental cost-effectiveness rate
HR+: Hormone receptor-positive
HER2: Human epidermal growth factor receptor 2-negative
MBC: Metastatic breast cancer
MAIC: Matching-adjusted indirect comparison
WTP: Willingness to pay
GDP: Gross domestic product
PSM: Partitioned survival model
